# Importance of crop phenological stages for the efficient use of PGPR inoculants

**DOI:** 10.1038/s41598-021-98914-9

**Published:** 2021-10-01

**Authors:** Alexandra Stoll, Ricardo Salvatierra-Martínez, Máximo González, Jonathan Cisternas, Ángela Rodriguez, Antonio Vega-Gálvez, Jaime Bravo

**Affiliations:** 1 CEAZA, Centro de Estudios Avanzados en Zonas Áridas, La Serena, Chile; 2 Instituto de Investigación Multidisciplinar en Ciencia y Tecnología, Universidad de la Serena, La Serena, Chile; 3 Programa de Doctorado en Biología y Ecología Aplicada, Universidad De La Serena, La Serena, Chile; 4 Departamento de Ciencias Básicas, Facultad de Ciencias Básicas, Universidad Santo Tomás, Ruta 5 Norte 1068, La Serena, Chile; 5 Departamento de Ingeniería en Alimentos, Universidad de la Serena, Av. Raúl Bitrán 1305, Casilla 599, La Serena, Chile; 6 Microbiología Aplicada e Innovación Agroalimentaria, Universidad Tecnológica de la Costa, Stgo Ixcuintla, Nayarit México

**Keywords:** Field trials, Plant biotechnology, Applied microbiology

## Abstract

During the last decades, the incorporation of beneficial microorganisms in agriculture crop management has become a common practice. Seed coating of these microorganisms still faces technical issues, which limit its implementation in conventional agriculture. An adaption to widely established agricultural practices, e.g. fertigation, could help to overcome these issues. Here, using *Bacillus velezensis* strain BBC047, we show the influence of the crop phenological stages on the efficiency and success of microbial inoculation under agricultural conditions. In the commercial nursery, strain BBC047 improved growth in a variety of horticulture crops like basil, cabbage, tomato and bell pepper, the latter with the strongest effects in strengthening and accelerating the seedling growth (root and aerial biomass). For a field trial under productive conditions, different application strategies were compared, using bell pepper (*Capsicum annuum* L.) as crop under fertigation: conventional management (T1), application to the seedling (only nursery, T2), only post-transplant application (field, T3) and a combination of both (T4). In T2 and T4, the post-transplantation survival rate (p < 0.05) improved and the productivity of the plants increased (> 100%). Applications of BBC047 post-transplantation (T3) caused a lower increase in productivity (25%). Fruits from all three application strategies contained significantly more Vitamin C. We conclude that in conventional agriculture, the applications of PGPR inoculants to early crop phenological stages like nurseries are a viable alternative for the efficient use of PGPR inoculants. In comparison, a late introduction of a PGPR reduces its beneficial effect on crop productivity. We highlight that an appropriate timing in the use of PGPR inoculants is crucial for product development and success in sustainable agriculture.

## Introduction

During the last decades, the incorporation of beneficial microorganisms in agriculture crop management has become a common practice. Especially plant growth promoting rhizobacteria (PGPR^1^), are considered an alternative to reduce the use of synthetic fertilizers and pesticides and therefore the contamination of agroecosystems^2, 3^, due to their ability to adapt and colonize plant roots quickly in this environment. Within this heterogenic group of PGPR, the genus *Bacillus* spp. has attracted most attention, being considered a secure and rentable strategy to improve crop production^4^. Beyond their PGPR potential, the *Bacillus* spp. are able to resist desiccation, heat or UV radiation as spores, extending the viability of potential commercial products^5, 6^.

Nonetheless, the application of PGPR based biostimulants or biopesticides in field encounters several technical and biological constrains, such as viability of the PGPR strains, host plant compatibility, inoculum density or inoculation method^2^. Seed coating is a promising method for PGPR inoculation, but is still mainly focussed on protective treatments (e.g., pesticides) or seed bulking^7^. Additionally, for PGPR coating, various challenges, e.g. scaling up from the laboratory to the field, the formulation (stabilization of the microorganism, coating materials) need to be addressed to enable a wider use of seed coating in sustainable agriculture^8^.

For liquid or encapsulated application, technical aspects like the inoculation method and their importance for the establishment and permanency of inoculated PGPR in the host rhizosphere are well understood^9, 10^, as well as their correlation with the growth promotion effect^11^. While these aspects are extensively addressed by science, much less is known about the influence of the crop phenological stages on efficiency and success of PGPR inoculation^2^.

Against this background, our purpose is to define which phenological stage of the crop is most adequate for PGPR inoculation in agriculture production, using *Bacillus* spp. and several horticulture crops, e.g. basil, tomato, bell pepper. We compared three strategies of liquid application, injected to fertigation under productive conditions: (1) application to the seedling; (2) post-transplant application; and (3) a combination of both. As parameters for the effectiveness of these inoculations, we evaluate commercially important parameters for quality of nursery production as well as yield of greenhouse production.

## Material y methods

### Bacterial strain

For the bacterial treatments the strain BBC047 from the collection of the Laboratory of Applied Microbiology at CEAZA (Chile) was applied. This strain belongs to the species *Bacillus velezensis* (formerly *Bacillus amyloliquefaciens*) and was isolated in the Coquimbo Region from lettuce rhizosphere. Its main characteristics include its biocontrol capacity against several fungal phytopathogens, such as *Botrytis cinerea*, and the production of IAA^12^. Strain BBC047 was cultured in LB medium for 36 h at 30° C and 150 rpm, until reaching a concentration of ~ 10^9^ CFU/ml. Bacterial cells were collected and resuspended in sterile water, adjusting concentration to ~ 10^6^ CFU/ml for seedling inoculation or ~ 10^7^ CFU/ml for applications during transplantation and post-transplant.

### Nursery experiment with horticultural crops

For the nursery experiment six common horticultural crops were used: lettuce (*Lactuca sativa* L, cv Corona), broccoli (*Brassica oleracea* var. *italica* Plenck, cv Imperial), cabbage (*Brassica oleracea* L, cv Rinda), basil (*Ocimum basilicum* L, cv Italiana), tomato (*Solanum lycopersicum* cv. Cal Ace), and bell pepper (*Capsicum annuum* L. cv. “Correntin”). For each crop, 2 plug trays (420 cavities for lettuce, 240 cavities for the other crops) of a commercial sowing on peat as substrate were selected randomly and strain BBC047 was applied as follows: the first application when the first pair of true leaf were extended, a second and third application was carried out each separated by 7 days. In each application, approximately 500 ml per plug of the bacterial solution of ~ 10^6^ CFU/ml was used. The other plug trays of the commercial sowing per crop were considered "control". Seedlings were maintained in the horticulture nursery Servicios y All-macigos S.A., located in Coquimbito (Valle del Elqui) a 11 km de La Serena (S 29°54′27.92″, W 71° 9′41.17″). The trial was carried out under the management established in the nursery company (for details regarding irrigation, fertilization and pest management program see Tables S1).

Evaluation of growth parameters was carried out when seedlings reached transplant size (lettuce 30 days, broccoli 45 days, cabbage 45 days, basil 30 days, tomato 35 days, bell pepper 35 days). For each crop, the height, root length, aerial and root fresh weight of 20 seedlings (randomly picked) per treatment were quantified.

### Bell pepper farming experiment

In the greenhouse of the farming and commercial company All-Fresh Ltda, located in Coquimbito (Valle del Elqui) a 11 km de La Serena (S 29°54′27.92″, W 71° 9′41.17″), the bell pepper farming experiment with 4 treatments was installed. With a completely randomized design, where each treatment was composed by 4 replicates with 10 plants each. Treatments were established as follows: T1 = control, plants without application of the bacteria; T2 = plants with application only in the nursery and not after transplanting; T3 = plants with application of the bacteria from transplant to the first harvest; T4 = plants with application of the bacteria from nursery to the first harvest.

During transplantation, seedlings for treatment T3 and T4 were immersed for 1 h in a solution of strain BBC047 (~ 10^7^ CFU/mL), the others in seedlings in water (T1 and T2). The seedlings of the previous trial were transplanted to the greenhouse soil, whose physicochemical characteristics are presented in Table 1. The plantation density was 33,000 plants/hectare.

**Table 1:** Physical–chemical soil parameters of the bell pepper greenhouse.

Parameter	Unit	Average ± SD
EC	mS·cm^−1^ 25 °C	9.09 ± 0.08
pH		7.07 ± 0.02
Calcium	mg·L^−1^ Ca^2+^	587.18 ± 5.08
Magnesium	mg·L^−1^ Mg^2+^	106.11 ± 1.33
Potasium	mg·L^−1^ K +	635.50 ± 11.26
Sodium	mg·L^−1^ Na^+^	968.72 ± 10.80
Chlorine	mg·L^−1^ Cl^−^	265.95 ± 13.45
Sulfate	mg·L^−1^ SO_4_^2–^	2463.93 ± 25.50
Nitrate	mg·L^−1^ NO^3−^	2048.66 ± 34.80
Bicarbonate	mg·L^−1^ HCO^3−^	128.70 ± 2.61
Phosphate	mg·L^−1^ H_2_PO^4−^	158.27 ± 3.39
Boron	mg·L^−1^ B	5.23 ± 0.06
N-NO_3_ available	mg·L^−1^ N	83.68 ± 9.10
P-available	mg·L^−1^ P	16.75 ± 0.18
K-available	mg·L^−1^ K	939.84 ± 7.18
Organic matter	MO %	1.79 ± 0.02

The treatments T3 and T4 received 2 more bacterial applications via irrigation, 30- and 60-days post-transplant. Per plant, 50 mL of a bacterial solution with 10^7^ CFU/ml were administered 30 days post-transplant and 100 mL 60 days post-transplant. The trial was conducted under the management established in the agricultural company (for details regarding irrigation, fertilization and pest management program see Tables S1).

#### Growth parameters

In the nursery, the height of the seedlings was evaluated 21 days after sowing (second application). At the time of the transplant, height, leaf area, fresh weight of root and aerial part of 20 seedlings per treatment were quantified.

Post-transplant to the greenhouse, evaluations were made according to the phenological state of the plant, measuring the height of plants from the stem to the bud on the highest branch. At the beginning of flowering, the fully open fertile flowers were counted, excluding floral buds and already fertilized flowers. During the fruit set those fruits formed with a diameter > 2 cm were counted, excluding freshly fertilized flowers. In addition, the survival rate per treatment was recorded 22 days after transplantation. Evaluations were carried out in 40 plants per treatment (corresponding to 4 replicates with 10 plants each) until the end of the trial.

For time reference we use DPI (days post- inoculation, referring to the first inoculation realized to seedlings in the nursery).

#### Growth kinetics

Using the height data set, three mathematical models were applied to characterize the growth dynamics in each treatment (Gombertz, Logistic, and Weibull Model, details in Table S2), selecting that model with the best fit. Analyses were performed with the statistical package SigmaPlot version 11.0. (Systat Software, Inc., San Jose California USA, www.systatsoftware.com). The goodness of fit of each model was evaluated by statistical tests that include the sum of squared error (SSE), chi-square (χ2) and the root of the mean square error (RMSE) where the lowest values of SSE, χ2 and RMSE were considered (Table S2). The studies of the parameters were performed in triplicate.

#### Yield

During the trial, 4 times fruits with commercial size and quality (parameters: length, diameter and color) were harvested. In each harvested fruit, length, diameter (both measured with digital Caliper brand Mitutoyo, Japon) and weight were registered. The first harvest considered fruits "in green" and the three remaining "in red" with 80% to 100% of surface turned red. From the first harvest (= harvest 2) "in red" 10 fruits per block were randomly selected for laboratory processing and determination of vitamin C.

#### Vitamin C content

The content of vitamin C was determined as ascorbic acid, in fruits of the first "red" fruit harvest, according to Islam et al.^13^ with minor modifications. From each treatment, 10 fruits were lyophilized. Subsequently, in a precipitated beaker, 1.0 g of lyophilized fruit, 0.2 g of oxalic acid (Fluka, Germany) and 30 ml of deionized water were added. The volume obtained was homogenized in Ultraturrax (IKA, model T18, Staufen, Germany) at 9000 RPM for 2 min, then 20 mL of diethyl ether was added (Merck KGaA, Darmstadt, Germany). Finally, the mixture was homogenized for 30 s and centrifuged at 4472 g for 15 min at 4° C.

Then, a 5 mL aliquot of the aqueous phase was taken and mixed with 5 mL of a 4% w / v solution of potassium iodide (KI, Sigma Aldrich, Saint Louis, USA), 2 mL of a solution of acetic acid 10% v / v (CH3COOH Merck KGaA, Darmstadt, Germany) and three drops of a 1% starch solution prepared at the time. Afterwards, a titration was carried out with NBS (N-Bromosuccinimide; C_4_H_4_BrNO_2_, Merck KGaA, Darmstadt, Germany), which was prepared by dissolving 0.2 g of NBS in 1.0 L of deionized water. At the same time, a standard vitamin C solution (Fluka, Germany) was prepared by weighing 51.12 mg of the standard and 2.0 g of oxalic acid, which were dissolved in 250 mL of deionized water. The solutions remained in darkness during their use. The results were expressed in mg Vitamin C / 100 g DW (mg of Vit C / 100 g of dry matter).

### Statistical analysis

The comparisons between treatments during the nursery stage were carried out using the Student t test. The statistical analysis of the greenhouse data was performed by one-way ANOVA for the normal data, applying the Kolmogorov–Smirnov test. Subsequently a Fisher LSD test was used for the comparison of means. When the distribution of the data was not normal, the Kruskal–Wallis test was used and later a multiple comparison test of the Agricolae package in R. All analyses were performed using the Agricolae package in R^14^.

### Ethical statements

The study was carried out complying with local and national regulations. In this study was carried out in collaboration with the mentioned farmers, who permitted us to work in their property, installations (nursery, greenhouse) and collect samples from their production (seedlings/ fruits). All plant material was provided by these farmers. No additional permissions or licenses were required.

## Results and discussion

### Nursery experiment with horticultural crops

**Figure 1:**
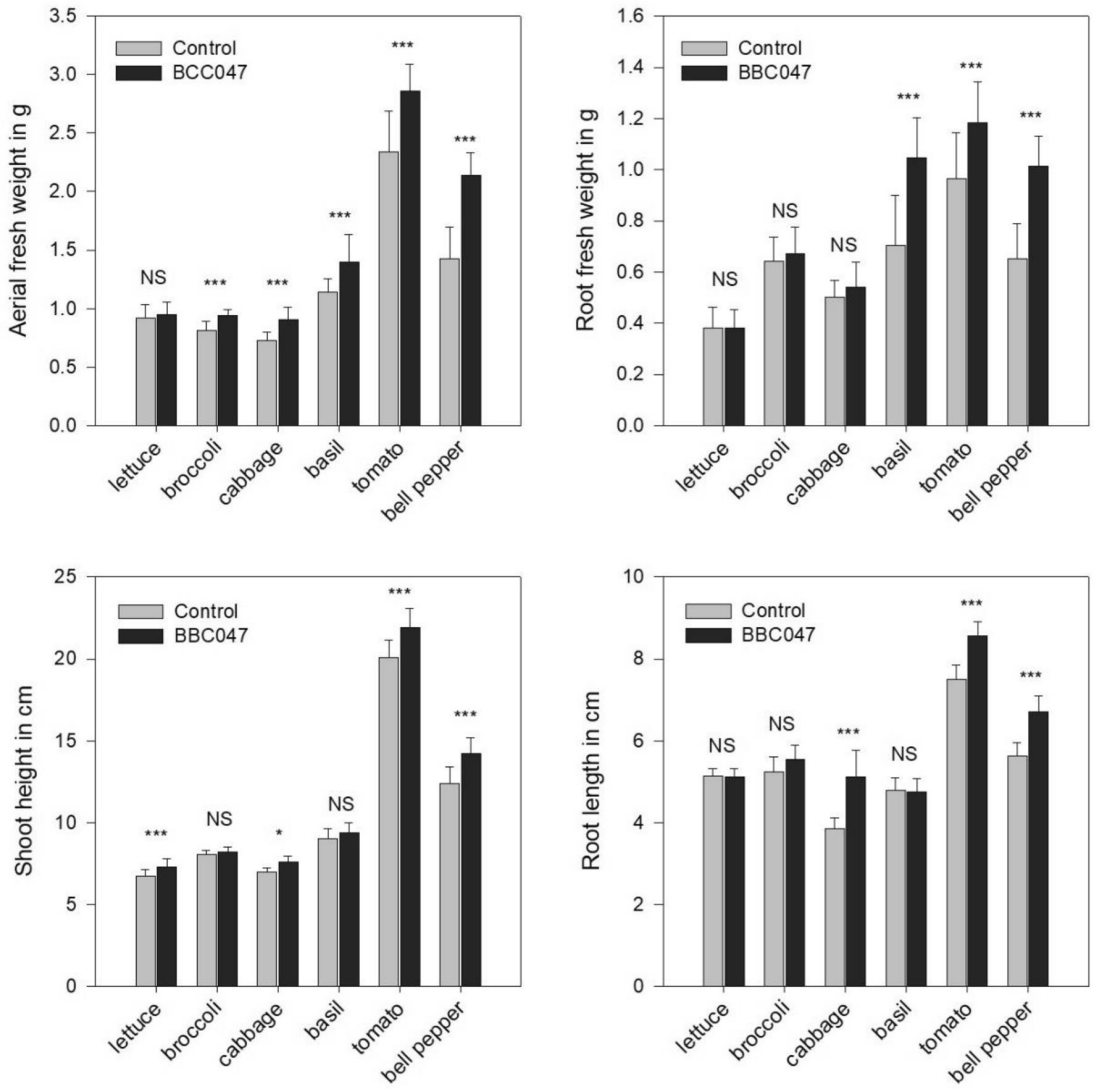
Effect of *B. velezensis* BBC047 application on five crop seedlings in horticulture nursery (at age of transplant, N = 20). Measured parameters: aerial fresh weight, root fresh weight, shoot height and root length. Statistical difference determined with ANOVA (*p < 0.05; ***p < 0.001; NS = not significant).

In the horticultural nursery experiment the five selected crops showed varying responses to the application of BBC047 (Fig. 1). In lettuce and broccoli only one parameter of aerial growth increased significantly (height and aerial fresh weight, respectively). For cabbage, basil, tomato and bell pepper both, root and aerial growth were improved. Similarly, Meng et al.^15^ described variable growth promotion results for *Bacillus velezensis* strain BAC03, inoculated to 8 different vegetables (beet, carrot, cucumber, pepper, radish, squash, tomato, and turnip). At nursery stage, the stimulation of root growth is important to prevent transplanting stress^16, 17^. In this sense, the significant increase of root biomass (basil, tomato, bell pepper) and root length (cabbage, tomato, bell pepper) are promising indicators for plant fitness and quality parameter of high relevance for the industrial production of seedlings. This growth promotion capacity is often attributed to the production of the phytohormones (like IAA or gibberellins) by the bacterial strain^12, 18^. Previously, Salvatierra et al.^12^ reported the production of the IAA phytohormone (16.64 + 1.98 μg / ml) by strain BBC047.

Particularly, the enhanced root development (tomato: + 22.7% root fresh weight, 14.8% root length; bell pepper: + 57% root fresh weight, 21.8% root length) are expected to contribute to the recovery of these seedlings after transplant and in their early growth and flowering^19^.

Similar to Torres et al.^20^, the strongest growth promotion was observed for Solanaceae (tomato, bell pepper). The most consistent response to applications of strain BBC047 were obtained in bell pepper, therefore this horticultural crop was chosen for the farming experiment.

### Bell pepper farming experiment

**Figure 2:**
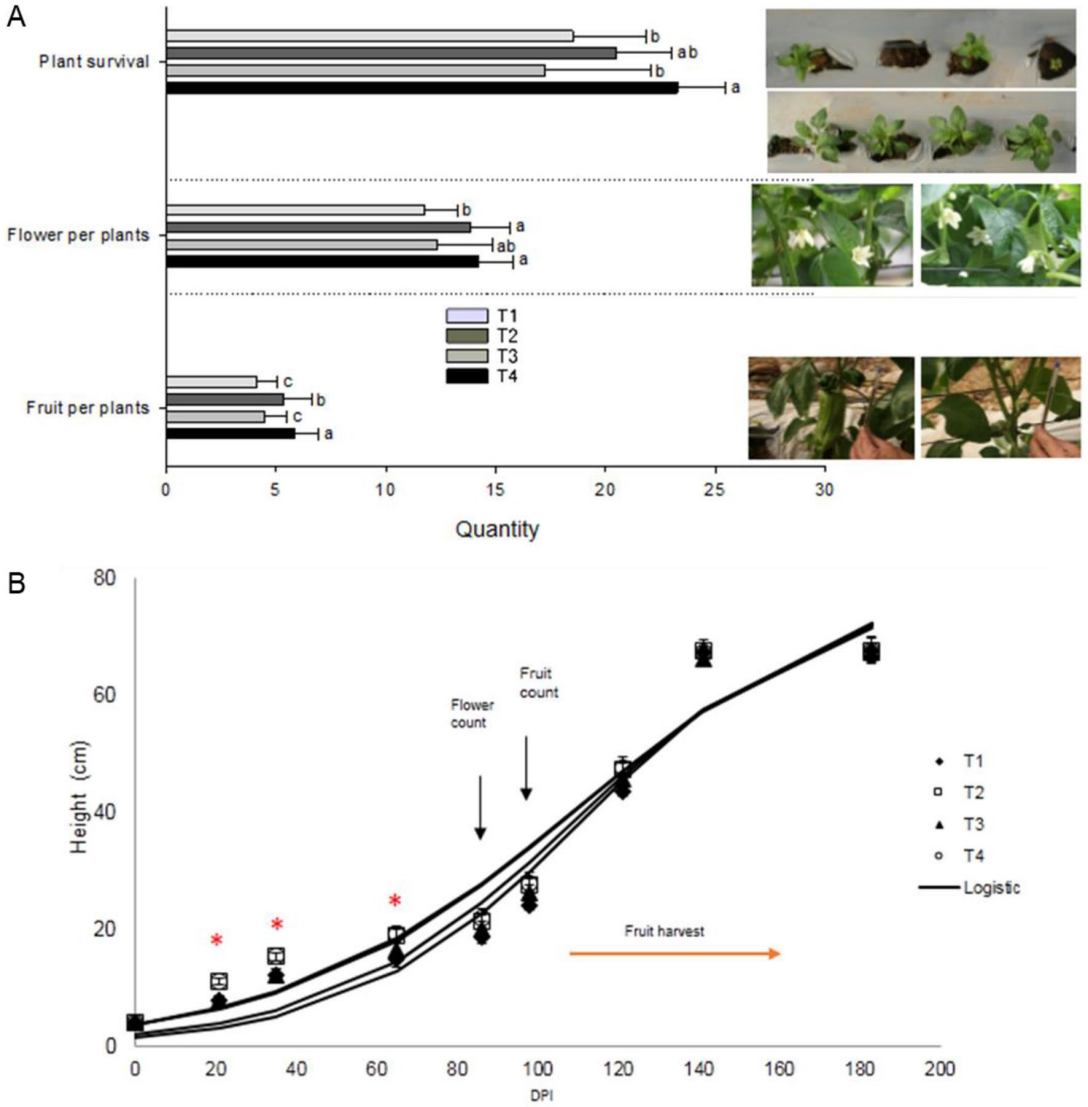
Effect of *B. velezensis* BBC047 application on bell pepper plants in productive greenhouse. (**A**) Plant survival, flowers and fruit set per plant (N = 40); plant survival evaluated at 57 DPI (3 weeks after transplant) according to ANOVA (*p* < 0.05) and Post-Hoc- LSD Fisher test, whereas flowers at 65 DPI and fruit set at 86 DPI were analyzed with Kruskal–Wallis test; photographs by R. Salvatierra-Martinez. (**B**) Growth kinetics of plant height; the model was generated using the average height at different phenological stages: in nursery at 21 and 35 DPI (N = 20); in greenhouse 65 to 183 DPI (N = 40). (*) represents statistical difference between treatments (p < 0.05).

After transplant to the productive greenhouse, the application of strain BBC047 from nursery (T2 and T4) improved post-transplant survival (Fig. 2A), which is consistent with that reported in other strains of *B. velezensis* (formerly *B. amyloliquefaciens*)^21^.

Growth kinetics: The analysis of the registered growth data (height) resulted in a good fit with the Linear, Gomperzt, Weibull and Logistic model, used to describe the growth kinetics (Table S2). The Logistic model best adjusted to the kinetics in all treatments, similar to the results of an irrigation trial with bell pepper under optimal irrigation reported by Demirel et al.^22^. In our experiment, during the first month post-transplant, plants of the treatments T2 and T4 (bacterial application since nursery) maintained their differences in the height compared to control (Fig. 2B). No significant effect of the bacterial application after the transplant (T3, Fig. 2B) was observed. However, one month after transplant the differences of height between all treatments diminish (no statistical difference), as plant physiologically passes from the vegetative growth state (establishment) to the first flowering period^23^.

Flowering and fruit set: Treatments T2 and T4 presented an increased number of flowers and fruit set, whereas for T3 no significant difference to control was registered (Fig. 2A). These results are consistent with Panayotov et al.^23^, suggesting that the benefits of the established plant–microbe-interaction were redistributed to improve reproduction. The missing effects in T3 might be explained with a lower colonization density of BBC047 strain reached in this treatment since transplant, versus T2 and T4, which were colonized by the same strain since early root development in nursery. Results of other authors^2, 24, 25^ and different crops are consistent with our findings.

**Figure 3:**
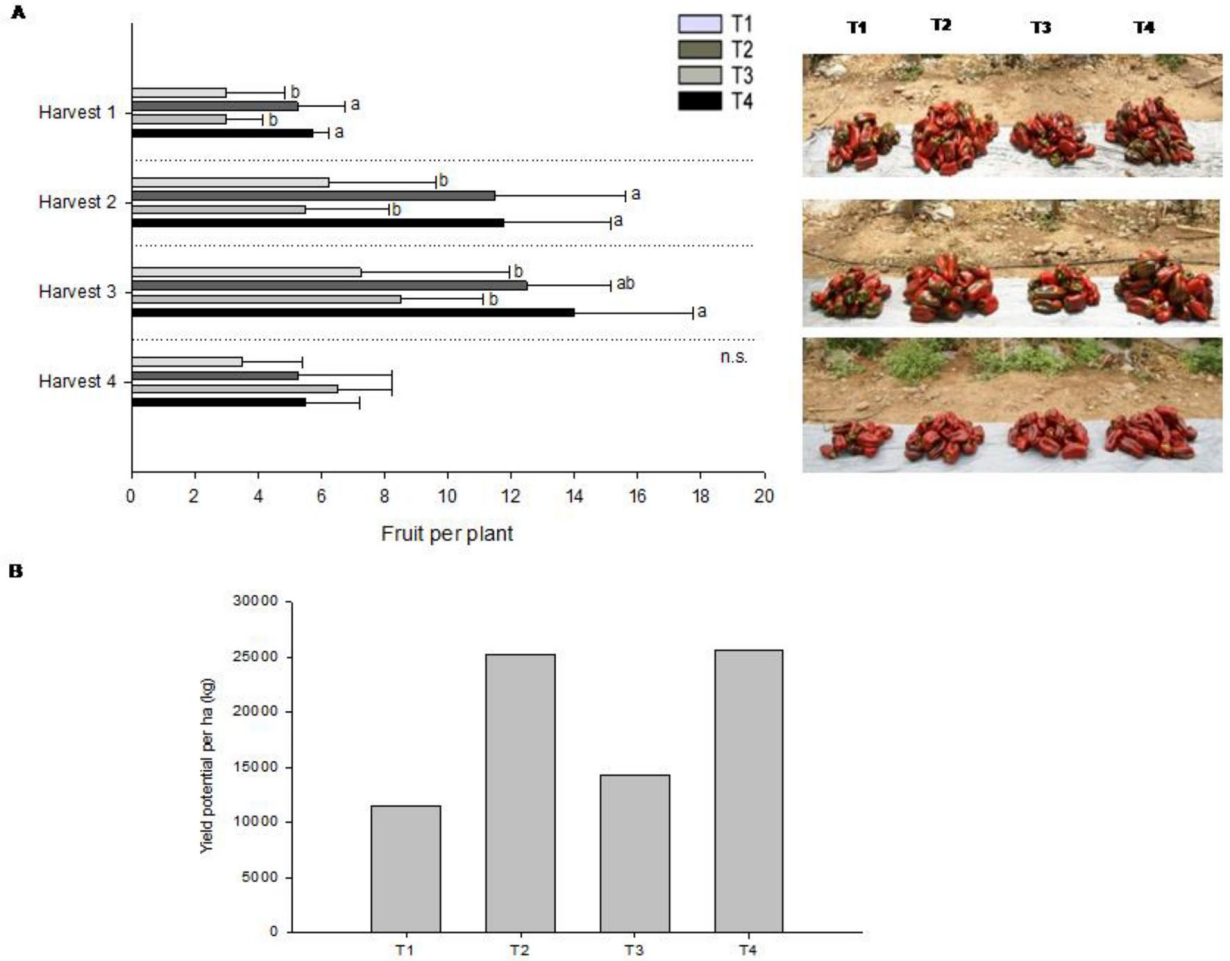
Effect of *B. velezensis* BBC047 application on bell pepper productivity and yield in productive greenhouse. (**A**) Fruit production per treatment in four representative harvests (bars indicate average of harvested fruits per plant and treatment, statistical differences determined with ANOVA (*p* < 0.05) and Post-Hoc-LSD Fisher test), photographs by R. Salvatierra-Martinez; pictures right: piled fruits from one replicate per treatment and harvest. (**B**) Extrapolation of the yield obtained per treatment in tons/hectare.

Yield: The plant density in this trial was 3.3 plants/m^2^, expecting an average of 15.6 to 22.75 marketable fruits per plant^26^. Our control treatment (T1) produced slightly less fruits per plant (14.8). In contrast, during the first three harvests, for the treatments T2 and T4 a significantly higher number of marketable fruits was registered (31.5 each). For the last harvest the number of fruits was not significantly different among the treatments (Fig. 3A). However, post-transplant bacterial applications (T3) did not show significant differences with respect to the control T1. Fruit quality parameters, as size and weight were not affected by bacterial treatments, which coincides with that reported by Panayotov et al.^23^.

The average yield of bell pepper is very variable, ranging between 49.4 and 178.4 t/ha, depending on e.g. plant density, variety and soil fertility^26, 27^. The productivity of the control treatment (73.50 t/ha) corresponds to this range (Fig. 3B). The obtained yield from all bacterial treatments is higher. Nonetheless, for treatments T2 and T4 the yield increased twice (161.7 and 163.9 t/ha) compared to T1, which represents the crop under conventional management conditions (Fig. 3B). The post-transplant bacterial applications (T3) also increased total yield to 92.4 t/ha (approx. 25% increase versus T1).

**Figure 4:**
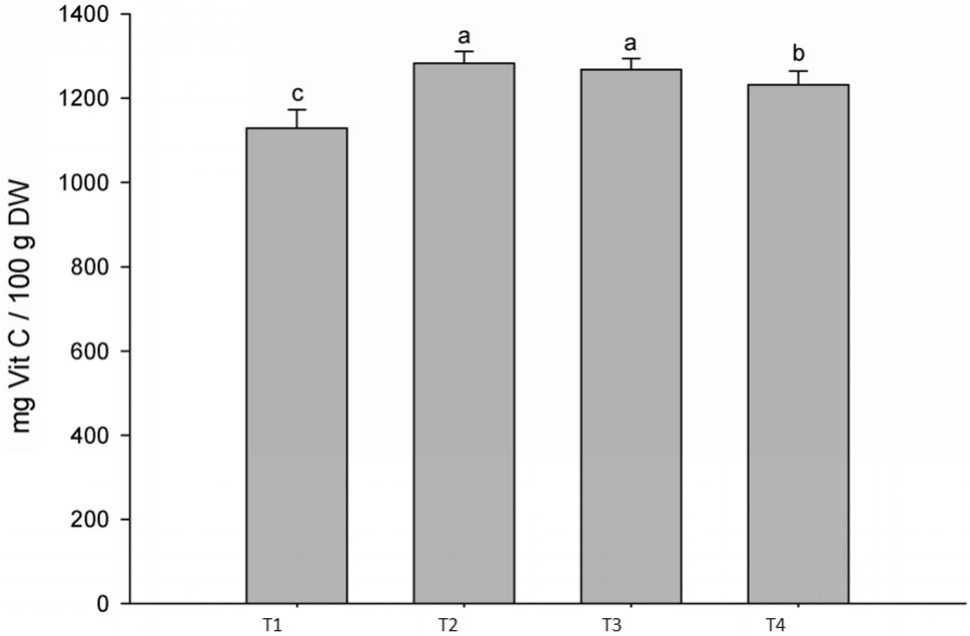
Content of vitamin C in fruits collected in the first “red” harvest (= harvest 2).

Vitamin C content: All bacterial treatments generated a significant increase in the vitamin C content in the fruits of the first harvest in red (Fig. 4). These results add new information regarding the ability of strain BBC047 to alter the biochemical composition and improve antioxidant capacity of bell pepper, enhancing the value of these fruits as functional food^28, 29^. Similar results were reported by Rezvani et al.^30^ and Nahed et al.^31^ for bell pepper and relative crops like tomato^32^. The involved metabolic mechanisms of the plant remain unclear, but should be investigated in future.

Overall, both treatments (T2 and T4) applied on seedling in nursery resulted most effective, whereas dipping on transplant and later applications barely increased crop productivity. In this study for the first time, the phenology of the plant is recognized as a crucial factor for an effective PGPR application in an agronomical context. We highlight that at early phenological stages of crop development in the nursery, the best conditions for colonization and long-lasting establishment of the PGPR in rhizosphere microbial community are given.

Different factors could be contributing to the successful colonization of strain BBC47 in the early development stages, beyond the application of a highest concentration of bacteria than the present in the bulk soil. Changes in the root bacteria communities are regulated by the plant, through exudation of organic compounds at the different phenological stages. According to the nature (sugar, sugar alcohols, plant hormones, nucleotides, etc.) and quantity of these compounds, it will determine the composition of the microorganisms associated to the plant root at the different development stages, where sugars as sucrose have been described as an important factor for the development of symbiotic plant-microbial interaction^33, 34^. The above could determine, according to the specie that the microbial community can be more susceptible to incorporate a new microorganism in seedlings than other vegetative or reproductive development stages in response to the plant exudates. Thus, determine the specific dynamics of the root exudates for each crop could help to identify the best time for the PGPR inoculum application and improve our results in this and other crops.

In addition, seedlings regulate the composition of the rhizosphere with the purpose of improving their fitness in the early development stages, where particularly the root system is susceptible to primary colonization^35^. Chaparro et al.^33^, showed in *Arabidopsis* that the rhizosphere bacterial communities are clearly separated into two clusters: an early growth-stage community (seedlings) and a later one (vegetative, bolting and flowering), showing a significant shift of bacterial structure of the rhizosphere. At the same time, their meta-transcriptomics analysis of different phenological stages revealed that transcripts associated to pathogenic disease are significantly expressed in the first development stages^33^, when *Arabidopsis* is more susceptible to pathogenic diseases. Kwak et al.^36^ realized transplantation of rhizosphere microbiota from resistant plant to phytopatogenic fungi *Ralstonia solanacearum* to susceptible tomato plants, suppressing the disease symptoms in the last one. This result suggests that, based on the plant genetic traits, it not always can recruit bacterial allies to protect itself but can maintain it if it is supplied to their microbiome. In this context, based on their biocontrol capacity, the strain BBC47 could be recruited in the first development stages to protect pepper seedlings against phytopathogenic diseases. On the other hand, PGPR such as the strain BBC47 that produce auxin hormone (IAA) can induce the root branching in the seedling stage, structures that could improve the nutrient uptake through the increasing of the total surface area of the plant root system^37^, enhancing their growth and yield in the plants treated with the strain BBC47 during the seedling stage. These results are in accordance with Bashan et al.^2^, Ruzzi and Aroca^38^, who highlight the advantages of nursery inoculation for transplanted crops and the need of further research as well as knowledge transfer to farmers.

## Conclusions

The *B. velezensis* strain BBC047 is an efficient PGPR, applicable to different crops like tomato and bell pepper. In horticulture nursery, the seedlings treated with BBC047, additionally to their improved fitness, met with the commercial parameters (e.g. root development) approximately 5 to 10 days before the untreated seedlings, depending on the crop. For production, this represents 15 to 25% reduction in time needed from sawing to commercializable seedling, allowing the nursery to increase the annual production for each crop (as at least one more sawing could be realized). Thus, a greater yield per surface and higher income by sales volume could be generated, in addition to reducing the costs in human resources and the use of agricultural inputs (fertilizers, herbicides and pesticides). In the greenhouse, the bell pepper yield was increased between 25 and 120% with the application program realized with BBC047. The size of increase depends on the phenological stage of the plant at the beginning of the application program, BBC047 applications starting at seedling stage doubled bell pepper yield. Nonetheless, a combined nursery—post-transplant application caused a very limited improvement in the bell pepper yield, compared the costs this may originate. Based on our results, we consider that BBC047 strain combines important characteristics to be employed as part of an integrated crop management.

Beyond that, we could validate the positive effect of carrying out PGPR applications at juvenile plants on crop yield in the agricultural context. Moreover, we demonstrate that PGPR application in early plant development stages (e.g. seedling), raise the effectivity of the PGPR inoculant. In the context of conventional agriculture, we highlight the importance of seedlings as phenological key stage for an efficient integration of PGPR applications into crop management programs. PGPR integrating management programs at the nurseries would imply important savings in application costs and volumes, as well as in time and human resources and an increased productivity, compared to applications in greenhouse or field.

## Supplementary Information

Supplementary Information.
