# Supplementary material for: Importance of crop phenological stages for the efficient use of PGPR inoculants
Source: Sci Rep. 2021 Oct 1;11:19548. doi: 10.1038/s41598-021-98914-9 (PMC8486824; doi:10.1038/s41598-021-98914-9)
Supplement: Supplementary file 1 — Supplementary Information. [file 41598_2021_98914_MOESM1_ESM.docx]

**Supplementary Table S1.** Irrigation, fertilization and pest management program

**Nursery experiment with horticultural crops** (as provided by Servicios y Almacigos SA)

|  |  | Fertilization (kg/ha) during nursery period | | | | | | |
| --- | --- | --- | --- | --- | --- | --- | --- | --- |
|  | Period (days) | N | K | Ca | Mg | S | P | Micro-elements |
| lettuce | 25 | 7,6 | 9,8 | 2,4 | 1,0 | 0,7 | 0,8 | 1,6 |
| broccoli | 40 | 17,5 | 17,2 | 9,6 | 3,0 | 4,8 | 1,6 | 5,8 |
| cabbage | 40 | 13,3 | 13,9 | 11,4 | 3,2 | 4,5 | 1,4 | 14,2 |
| basil | 25 | 4,8 | 8,7 | 1,5 | 1,3 | 0,6 | 0,5 | 1,0 |
| tomato | 30 | 10,4 | 12,9 | 6,0 | 2,6 | 1,6 | 1,0 | 3,1 |
| bell pepper | 30 | 11,8 | 15,9 | 3,5 | 2,1 | 0,9 | 1,2 | 2,9 |

Indicated time for nursery period refers to maintenance after germination (after automated sowing plugs are incubated for 5 days in a climate chamber with approx. 21 to 24°C for germination). Afterwards, fertigation was carried out every second day with approximately 7,000 L/ha, where nutrients were applied as indicated in the table as period total for each crop.

Pest management consisted in periodical applications of Boscalid + Piraclostrobina 600g/ha (fungicide) and Diazinon 4L/ha (insecticide) as indicated by the manufacturer instructions.

**Bell pepper farming experiment** (as provided by All-Fresh Ltda)

| Period (days) | 35-85 | 85-140* | 140-200* | 200-260 |
| --- | --- | --- | --- | --- |
| Irrigation (m^3^/day) | 9 | 7,5 | 6 | 12 |
| Fertilization (kg/ha) | | | | |
| Ca(NO_3_)_2_ | 200 | 200 | 100 | 50 |
| Mg(NO_3_)_2_ | 200 | 200 | 100 | 50 |
| KNO_3_ | 100 | 300 | 200 | 100 |
| K_2_SO_4_ | 0 | 350 | 350 | 100 |
| NH₄NO₃ | 400 | 300 | 0 | 0 |
| H_3_PO_4_ | 200 | 50 | 50 | 50 |
| Pesticides | | | | |
| Weed control | Before plantation: Trifluralin 1-2 L/ha, Herbadox 4-5 L/ha | | | |
| Fungicides | Following application instructions: Azoxystrobin + Difenoconazol 0.6L/ha; Chlorthalonil 2L/ha, Chlorthalonil + Azoxystrobin 1.5L/ha | | | |
| Insecticides | Diazinon 4L/ha | | | |
| Mean T greenhouse (°C) | 23.5 | 17 | 14 | 20 |

*winter period

Plantation in beds covered with plastic mulch.

**Supplementary Table S2.** Functions of plant growth rate.

| Model Name | Integral form | Differential form | Reference |
| --- | --- | --- | --- |
| Gompertz | *a*exp(-*b*exp(-*c*t)) | *abc*exp-(*c*t)exp(-*b*exp(-*c*t)) | Rodríguez, et al. (2011);  Zeide, (1993) |
| Logistic | *a*/(1+*c*exp(-*b*t)) | *abc*exp-(*c*t)(1+*c*exp(-*b*t)^2^) |  |
| Weibull | *a*(1-exp(-*b*(t*^c^*))) | *abc*t^(^*^c^*^-1)^exp-(*b*t)*^c^* |  |

| Statistical tests | Formula |
| --- | --- |
| sum of squared error (SSE) | $SSE=\frac{1}{N}\sum_{j=1}^{N} \left(MR_{ej}-MR_{cj}\right)^{2}$ |
| chi-square (χ2) | $\chi^{2}=\frac{\sum_{j=1}^{N} \left(MR_{ej}-MR_{cj}\right)^{2}}{N-z}$ |
| mean square error (RMSE) | $R^{2}=\sum_{j=1}^{N} \frac{\left(MR_{cj}-\overline{MR}_{ej}\right)}{\left(MR_{ej}-\overline{MR}_{ej}\right)^{2}}$ |
